# School Nutrition Personnel Perceptions of School Salad Bars before and after COVID-19

**DOI:** 10.3390/nu16040488

**Published:** 2024-02-08

**Authors:** Katlyn Garr, Ashley Mendoza, Suzanne E. Mazzeo, Hollie A. Raynor, Lilian de Jonge, Kristina L. Tatum, Bonnie Moore, Melanie K. Bean

**Affiliations:** 1 Department of Pediatrics, School of Medicine, Children’s Hospital of Richmond at Virginia Commonwealth University, Richmond, VA 23229, USA; 2 Department of Psychology, Virginia Commonwealth University, Richmond, VA 23284, USA; 3 Department of Nutrition, University of Tennessee, Knoxville, TN 37996, USA; hraynor@utk.edu; 4 Department of Nutrition and Food Studies, George Mason University, Fairfax, VA 22030, USA; edejonge@gmu.edu; 5 Real Food for Kids, Arlington, VA 22101, USA

**Keywords:** salad bar, COVID-19 pandemic, cafeteria personnel, school food environment

## Abstract

Many schools have salad bars as a means to increase students’ fruit and vegetable intake. School nutrition programs experienced drastic changes to the school food environment due to COVID-19. The aim of the current study was to understand cafeteria personnel’s experiences related to salad bar implementation before the COVID-19 pandemic and in the current school environment to inform efforts to enhance salad bar sustainability. Seven elementary schools (*N* = 30 personnel) installed salad bars prior to COVID-19; three of these schools (*n* = 13 personnel) re-opened salad bars after COVID-19. Cafeteria personnel completed surveys assessing their experiences with salad bars at both time points. Satisfaction with salad bar implementation and training was high pre- and post-COVID-19. Most agreed that salad bars increased students’ fruit and vegetable intake, yet had concerns about cleanliness and waste. Perceived job difficulty increased post-COVID-19 (*p* = 0.01), and satisfaction with student salad bar training decreased (*p* = 0.001). Additional staff support and greater student training were needed post-COVID-19. Overall, salad bars were viewed favorably; however, more challenges and lower satisfaction were reported following COVID-19. Increasing support for cafeteria personnel is needed for salad bar sustainability and improving the school food environment.

## 1. Introduction

Few children consume the daily recommended amounts of fruits and vegetables (F&V) [1]. This is concerning, as regular consumption of F&V is associated with a reduced risk of chronic diseases (e.g., cardiovascular disease, cancers, hypertension) [2] in the long term and can help children maintain a healthy weight [3]. Ensuring children have access to heathy foods is critical for their overall health. Children from racially and ethnically marginalized and/or low-income backgrounds have lower F&V access and consumption [4,5] and are at an increased risk for food insecurity, obesity, and related chronic illnesses compared to children in other groups [6]. Given that children receive up to half of their daily meals at school, the school environment represents an excellent setting to improve F&V access and consumption, particularly for children from marginalized backgrounds [7]. 

The National School Lunch Program (NSLP) [8] provides free or reduced-price lunch for students from low-income backgrounds and helps address racial and economic disparities in F&V access and consumption. Standards set by the Healthy Hunger-Free Kids Act (HHFKA) mandate that school meals must consist of at least one fruit or vegetable, with additional guidelines regarding vegetable variety (e.g., dark green, red/orange, legumes, starchy vegetables) [9]. Despite these promising policies (demonstrated to improve children’s diet quality [10]), students’ F&V intake remain suboptimal [1]. Salad bars are increasing in popularity as a means to help meet these standards and enhance F&V intake [11]. This enthusiasm is in part due to salad bars’ ability to offer a variety of F&Vs and foster choice, aligning with children’s increasing autonomy over their food choices and eating behaviors during this developmental period [12]. Thus, school salad bars might offer a unique opportunity to increase F&V intake, decrease caloric consumption, and reduce obesity risk [13,14]. However, to maximize the benefits of school salad bars, it is important that they are feasible and sustainable for school nutrition personnel—the individuals tasked with implementing salad bars and ensuring compliance with federal-level nutrition policies.

The COVID-19 pandemic significantly impacted the school food environment [15]. When schools re-opened, new regulations to ensure the safety and health of students were established (e.g., sanitization, social distancing) [16]. School meal mandates also changed rapidly, and multiple amendments were passed to allow school nutrition departments to adapt to the changing environment and supply chain disruptions. For example, a nationwide waiver was granted to allow flexibility in meal patterns and serving requirements to increase compliance with COVID-19 safety measures. These policy changes increased the daily work of cafeteria school personnel, at a time also marked by constrained resources [15,17]. Our prior research with school salad bars (pre-COVID-19) highlighted the potential that school environment factors (other than just the presence of a salad bar) might influence F&V intake, given the mixed findings related to student F&V consumption within schools with salad bars [18,19]. Although not evaluated in the prior study, one potential factor may be implementers’ perceptions and experiences with school salad bars, particularly when there are competing demands (e.g., nutrition requirements, infection prevention and control) such as after the COVID-19 pandemic. For example, prior to COVID-19, Bruening and colleagues reported that school nutrition managers who viewed salad bars positively were more likely to have salad bars installed in their school compared to school nutrition managers with less favorable views [20]. Salad bars are considered to be more effective in increasing student F&V selection and consumption when there is more buy-in and role modeling within the school [11]. Therefore, supporting school nutrition personnel who are implementing district- and federal-level nutrition policies and programs might increase salad bar buy-in and improve both salad bar implementation and the overall school food environment to support optimizing F&V intake. 

The goal of the current study was to describe school cafeteria personnel’s perceptions, experiences, and feedback related to salad bar implementation. We first describe responses from cafeteria personnel from schools that had salad bars before the COVID-19 pandemic. We then describe and compare cafeteria personnel’s perceptions of salad bar implementation before and after the COVID-19 pandemic using a subset of schools that reopened salad bars after the COVID-19 pandemic. We hypothesized that there would be less satisfaction and greater challenges and barriers to salad bar implementation following COVID-19.

## 2. Materials and Methods

### 2.1. Participants and Procedures

The current study is a secondary analysis of data from a large cluster randomized controlled trial (RCT), investigating the impact of school salad bars on student F&V selection and consumption in a large public school district in the Mid-Atlantic United States. See Bean et al., 2022 [21] for further details about the RCT. Salad bars were installed in seven elementary schools (K–6th grade) in 2018–19 and 2019–20. Data collection was paused in March 2020 when schools closed due to the COVID-19 pandemic. When schools re-opened in the fall of 2020, salad bars remained closed. In 2022–23, salad bars began re-opening. Three of the seven schools were reassessed. These schools were selected for reassessment because their salad bars re-opened within the study period.

The current study describes the satisfaction, perceptions, and feedback of cafeteria personnel from: (1) seven schools that opened salad bars prior to the COVID-19 pandemic and (2) three of the schools that re-opened salad bars following the COVID-19 pandemic. Cafeteria personnel completed paper surveys six weeks after the initial salad bar opening; the subset of schools that re-opened salad bars repeated the survey six weeks after re-opening post-COVID-19. Informed consent was obtained prior to starting the survey. Personnel could opt to enter a raffle for a $25 gift card to thank them for their participation. All study methods were approved by the institution’s review board.

### 2.2. Measures

Cafeteria personnel completed four sociodemographic questions assessing their school role (e.g., cafeteria staff, cafeteria manager), gender, race, and ethnicity.

Cafeteria personnel completed nine survey questions assessing satisfaction with various aspects of the salad bar and perceptions of the school food environment. Responses were based on a 4-point scale (Strongly Disagree, Disagree, Agree, and Strongly Agree), which was collapsed to a 2-point scale (Strongly Disagree/Disagree and Strongly Agree/Agree) for analyses due to small variation. Personnel also indicated how much each of the 11 barriers impacted their ability to run the salad bar or impacted their daily work (e.g., student behaviors, staffing, time, regulatory requirements) using a 3-point scale (Not at All, A Little, A Lot). Barriers were informed by consultation with Food and Nutrition services and review of the empirical literature. Lastly, personnel responded to open-ended items further assessing challenges and successes of salad bars and suggestions for improvement. Surveys were anonymous and available in personnel’s preferred language. This survey was adapted from our prior work within the school cafeteria environment [22] but has not been independently validated.

### 2.3. Data Analysis

Analyses were conducted using SPSS v28. Descriptive statistics were calculated for personnel characteristics (e.g., race, ethnicity, gender, school role) and survey responses (*N*, %) for the overall sample. Frequency statistics were also calculated for survey responses for schools that had salad bars both pre- and post-COVID-19. Given the small sample size, Fisher’s exact test (two-tailed) evaluated whether questions related to salad bar perceptions and satisfaction differed between pre- and post-COVID-19 for schools that had salad bars at both time points. Open-ended survey responses assessing program feedback were explored for general themes for schools that received salad bars both pre- and post-COVID-19. *p*-values < 0.05 indicated statistical significance.

## 3. Results

### 3.1. School and Personnel Characteristics

Across seven schools, three schools were Title I, and between 40% to 76% of students were from racially and/or ethnically marginalized backgrounds. NSLP participation ranged from 31% to 78%, with 9% to 62% of students eligible for free or reduced-price lunches. The survey response rate for cafeteria personnel was 89% (*n* = 30 pre-COVID [84%]; *n* = 13 post-COVID-19 [100%]). Respondents were mostly cafeteria staff (73%), and a majority were female (90%), Asian American/Pacific Islander (56%), and non-Hispanic (68%). All three schools that were reassessed after COVID-19 were Title I, and 70% to 78% of students were from racially and/or ethnically marginalized backgrounds. NSLP participation ranged from 52% to 63%, with 57% to 73% of students eligible for free or reduced-price lunches. Among these three schools, surveys were completed by 100% of personnel pre- (*n* = 15) and post-COVID-19 (*n* = 13). Eighty percent of personnel worked in the school at both time points. Sociodemographic characteristics were similar for both pre- and post-COVID-19 (*p*-values > 0.05). See Table 1 for further sociodemographic information for personnel at both time points.

### 3.2. Salad Bar Implementation before COVID-19

For the seven schools that installed salad bars before the COVID-19 pandemic, overall satisfaction with salad bar implementation was high (96%). Personnel were 100% satisfied with the salad bar training they and the students received. Personnel perceived that the salad bar increased students’ fruit and vegetable intake (97%) and that their school promoted healthy eating (100%). Approximately 35% of personnel perceived that the salad bar made their job more difficult, and 66% perceived that the salad bar increased food waste. Barriers and challenges included ensuring students’ trays met NSLP meal requirements (48%), keeping the salad bar clean (44%), and completing yield forms and temperature logs (47%).

### 3.3. Salad Bar Implementation before and after COVID-19 in Subset of Title I Schools

Overall satisfaction with salad bar implementation and personnel training was high pre- (100%) and post-COVID-19 (77%; no significant change; *p*-values = 0.098). At both time points, most personnel agreed that the salad bar increased students’ fruit and vegetable intake and that the school promoted healthy eating (100% vs. 85%; no significant change; *p* = 0.222 and *p* = 0.206, respectively). Perceived job difficulty due to the salad bar significantly increased following COVID-19 (14% [pre] vs. 62% [post], *p* = 0.018), and satisfaction with student salad bar training significantly decreased (100% [pre] vs. 46% [post], *p* = 0.002). At both time points, personnel liked that students had access to fresh fruits and vegetables (100% [pre] vs. 77% [post]; no significant change; *p* = 0.098), yet had concerns about food waste (57% [pre] vs. 85% [post]; no significant change; *p* = 0.209). See Table 2.

**Figure 1:**
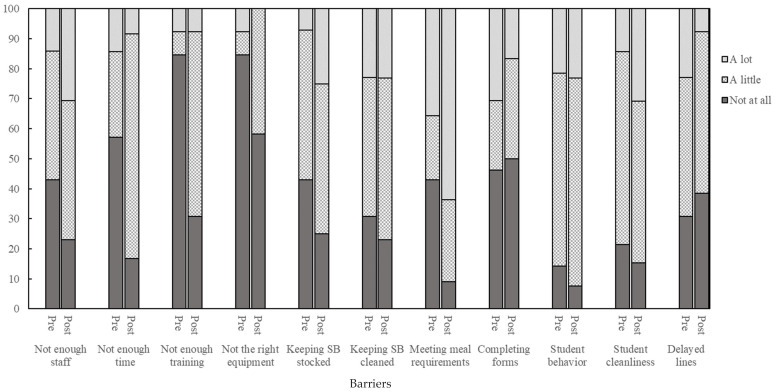
Cafeteria personnel’s ratings of how much specific barriers impacted their daily work for a subset of schools (*n* = 3) who had salad bars (SB) pre- (*n* = 15 personnel) and post-COVID-19 (*n* = 13 personnel).

Figure 1 shows how much of an impact (none, a little, a lot) different salad bar-related barriers had on cafeteria personnel’s daily work. Not having enough time became a greater barrier following COVID-19, with 29% of personnel reporting that it had “a little” impact on their daily work before COVID-19 compared to 75% after COVID-19. Making sure students’ trays met the minimum NSLP meal requirements also became more challenging after COVID-19 (36% [pre] vs. 64% [post] reporting “a lot” of impact). Completing the salad bar yield form and temperature log became easier for personnel following COVID-19; prior to COVID-19, 31% responded “a lot” of impact on their job compared to 17% post-COVID-19. Delayed cafeteria lines also improved following COVID-19, with 23% reporting “a lot” of impact on their job compared to 8% after COVID-19. Managing student behavior (21% [pre] vs. 23% [post] reporting “a lot” of impact) and keeping the salad bar clean (23% pre- and post-COVID-19 reporting “a lot” of impact) remained a barrier at both time points.

Personnel responded to open-ended questions assessing satisfaction, concerns, and areas for improvement for the salad bar. There were some similarities in salad bar themes among personnel pre- and post-COVID-19. At both time points, personnel described that preparation (e.g., organization, teamwork) was important to run the salad bar well, and they liked that the salad bar provided students access to fresh and healthy foods. For example, one personnel member reported, “kids who have been eating fast food can come eat fruits and vegetables”. Food waste and salad bar cleanliness were also concerns both before and after COVID-19. One personnel member stated that the “number of students increased [which] caused a shortage of food”. Post-COVID-19, personnel identified that additional staff support and greater student salad bar training were needed. Personnel described several ways to improve the salad bar and make it more successful, including “send videos and documents to parents”, “help students be aware of disease prevention”, and “remind kids every 3 months about [salad bar] roles”. See Table 3 for a full list of personnel’s responses.

## 4. Discussion

The current study sought to understand cafeteria personnel’s perceptions of and experiences with school salad bar implementation before and after the COVID-19 pandemic to inform strategies to enhance salad bar sustainability and, ultimately, student F&V in-take. To our knowledge, this is the first study to obtain post-COVID-19 perspectives from cafeteria personnel, individuals critical to the feasibility and effectiveness of school salad bars. The findings indicate that cafeteria personnel were satisfied overall with salad bar training and implementation and perceived the salad bars as beneficial to students’ health. However, it was evident that barriers and challenges with salad bar implementation increased following the COVID-19 pandemic. Increasing the feasibility and sustainability of salad bars will maximize their potential benefits related to improving student F&V intake. Results from the current study can be used to inform and tailor future programs aimed at supporting cafeteria personnel in their implementation of salad bars and nationwide nutritional policies within the NSLP, a strategy intended to optimize children’s dietary intake.

A consistent finding across both time points was the perception that salad bars increased access to F&V for students, including increased dietary choices, particularly for children who have reduced access to healthy foods. This is important given the HHFKA and NSLP initiatives to reduce disparities in F&V access and consumption [8,9]. Further, cafeteria personnel reported that salad bar implementation increased student F&V intake. Prior research has yielded mixed findings regarding the relation between school salad bars and increased student F&V consumption [19,23,24], and more research using objective measurements (e.g., plate waste studies) [25] of student F&V consumption is needed. Results from the current study also show that cafeteria personnel’s satisfaction and training are important for successful salad bar implementation. Preparation, communication, and organization were noted as valuable in helping cafeteria personnel run salad bars well. Including additional information on these aspects or incorporating teambuilding activities into trainings may improve salad bar implementation.

Across both time points, increased food waste and ensuring students’ trays met NSLP meal requirements were concerns for cafeteria personnel, and the COVID-19 pandemic exacerbated these challenges. Fruits and vegetables are often the foods wasted at school lunch [26], and addressing factors (e.g., dietary choice, portion sizes, product ordering) [27] linked to food waste will be important as school salad bar installations increase. Several personnel also noted the need for additional staff, particularly following the COVID-19 pandemic. Reopening schools during the COVID-19 pandemic presented unprecedented challenges, and adhering to rapidly changing school nutrition policies following the pandemic might have contributed to the increased salad bar barriers cafeteria personnel experienced. Prior research has shown that schools with greater school-level resources (e.g., full-service kitchen, food service companies) were more likely to have salad bars [20]; thus, increasing support and resources for cafeteria personnel will be important to lessen the burden of salad bar implementation. Many cafeteria personnel were unsatisfied with student salad bar training; thus, incorporating additional student training and refresher training can be used to address appropriate salad bar behavior, food waste, cleanliness, and infection prevention.

Strengths of the current study include data collection from multiple schools from an economically and racially diverse school district, obtaining data before and after the COVID-19 pandemic, obtaining perspectives from key stakeholders in salad bar implementation, and a high survey response rate. There are also limitations to consider when interpreting the current study’s results. The sample size was small and limited to cafeteria personnel from one district. A larger sample of cafeteria personnel and/or obtaining additional perspectives from other school staff and students could improve the understanding of salad bar feasibility and sustainability. Although surveys included open-ended questions, using semi-structured interviews might have allowed for increased understanding of the barriers that cafeteria personnel experienced. Surveys were completed six weeks after salad bars opened. Future research should evaluate if salad bar barriers change over longer periods of time (e.g., across the school year) as exposure and familiarity with the salad bar increase, and to understand the long-term sustainability of salad bars in elementary schools. It will also be important to evaluate if personnel perceptions of salad bars relate to objective measurements of student F&V selection and consumption.

## 5. Conclusions

Cafeteria personnel were overall satisfied with school salad bars, yet the COVID-19 pandemic had a significant negative impact on cafeteria personnel’s daily workload and salad bar implementation. School salad bars are highly promoted due to their potential to increase student F&V intake and foster dietary choice [13]. As salad bars continue to re-open post-COVID-19 [28], it will be important to support cafeteria personnel with salad bar implementation. Further, school nutrition policies and programs are constantly changing and developing, including the pending proposed nutrition guidelines by the U.S. Department of Agriculture [29]; understanding the needs of cafeteria personnel to carry out such changes will be critical. Research on school salad bars is limited, [30] and more research is needed to explore other factors, in addition to cafeteria personnel’s experiences, that influence salad bar feasibility and sustainability and student dietary intake.

## Figures and Tables

**Table 1:** Sociodemographic factors of cafeteria personnel from seven schools in a mid-Atlantic region that had salad bars installed pre-COVID-19, and from the subset that had salad bars pre- and post-COVID-19.

	All Schools (*N* = 7)	Subset of Schools (*n* = 3)	
Characteristic	Pre-COVID-19 (*N* = 30 Personnel)	Pre-COVID-19 (*n* = 15 Personnel)	Post-COVID-19 (*n* = 13 Personnel)
School Role, *n* (%)			
Cafeteria Staff	19 (73.1)	7 (63.6)	8 (66.7)
Cafeteria Manager	7 (26.9)	4 (36.4)	3 (25.0)
Other	-	-	1 (8.3)
Race, *n* (%)			
Asian American/Pacific Islander	15 (55.6)	9 (64.3)	7 (53.8)
Latinx	8 (29.6)	4 (28.6)	3 (23.1)
African American/Black	1 (3.7)	-	1 (7.7)
Caucasian/White	1 (3.7)	-	-
Other	2 (7.4)	1 (7.1)	2 (15.4)
Ethnicity, *n* (%)			
Non-Hispanic	19 (67.9)	10 (66.7)	9 (69.2)
Gender, *n* (%)			
Female	27 (90.0)	13 (86.7)	12 (92.3)

Note. Categories that do not equal 100% are due to missing data.

**Table 2:** Cafeteria personnel’s ratings of satisfaction and perceptions about salad bars for the subset of schools (*n* = 3) who had salad bars before and after COVID-19.

	Pre-COVID-19 (% Agree/Strongly Agree) ^a^	Post-COVID-19 (% Agree/Strongly Agree) ^a^	*p* ^b^
Satisfaction with:			
Personnel training	100	76.9	0.098
Student education	100	46.2	0.002
Ensuring students’ trays met meal requirements	100	76.9	0.098
Overall salad bar implementation	100	76.9	0.098
Perceptions of:			
School cafeteria promotes healthy eating	100	84.7	0.206
Salad bar made job more difficult	14.2	61.6	0.018
Glad students have salad bars	100	76.9	0.098
Salad bars increased waste	57.2	84.7	0.209
Salad bars increased student fruit and vegetable intake	100	84.6	0.222

^a^ Responses were collapsed from a 4-point Likert scale (Strongly Disagree, Disagree, Agree, Strongly Agree) to Disagree/Strongly Disagree and Agree/Strongly Agree. ^b^ *p*-values based on Fisher’s Exact Test.

**Table 3:** Cafeteria personnel’s responses to open-ended questions assessing salad bar feedback for the subset of schools (*n* = 3) who had salad bars both before and after COVID-19.

Questions	Responses	
	Pre-COVID-19 (*n*)	Post-COVID-19 (*n*)
What do you like about the salad bar?	Fresh foods/fruits/veggies (10) Students have options (2) Children like what they eat (1) High quality meals (1)	Fresh/healthy foods/fruit/veggies (7) Did not like/students did not eat (2)
What helps you run the salad bar well?	Organization, preparation, teamwork (5) Training (2) Problem-solving (1) Communication w/students (1)	Task assignment, cleaning (3) Adding more staff (2) Teaching students (2) Having a good spirit (1)
What concerns do you have about the salad bar?	Cleanliness (4) Food waste (2) Student behavior/respect (2) Food shortage (1)	Food waste (3) Cleanliness (2) Foods selected indiscriminately (2) Number of dishes (1)
What do you wish you knew or understood better before your salad bar opened?	Be prepared, organized (6) More staff/student training (2) Satisfied with training received (2)	More information, training (3) The level of task difficulty (1) Order less food (1)
What was the most valuable part of the training you received before your salad bar opened?	Videos, documents, PowerPoints (4) Preparation, cleaning (4) Visiting another school with a salad bar (2) Real-time training (2)	All training, videos, documents (6) Setting up, preparation (4) Cleaning, avoiding infection (3) Nothing (1)
What other training or resources would help you run the salad bar better in the future?	More training (4) Cutting fruit (1) Understanding amount of fruit (1) Organization (1) Increased staff communication (1) Satisfied with training received (1)	Visit a school with a salad bar (2) Satisfied with training received (2) More student training (1) Cleanliness (1)
What ideas do you have about how to make the salad bar more successful?	Offer more fruits, veggies (3) Satisfied as is (2) Additional staff (1) Improve staff communication (1) More student training (1) Involve parents (1)	Additional/engaged staff (3) More student training, booster trainings (3) More time (1)

## Data Availability

The raw data supporting the conclusions of this article will be made available by the authors on request.
